# Machine learning-enhanced causal inference of surgical decisions and rehabilitation strategies in traumatic brain injury

**DOI:** 10.3389/fneur.2025.1685335

**Published:** 2025-11-13

**Authors:** Elyas Irankhah, Madhavi Pagare, Lokesh Chetla, Jiabin Shen, Mohammad Arif Ul Alam, Kelilah L. Wolkowicz

**Affiliations:** 1 Department of Mechanical and Industrial Engineering, University of Massachusetts Lowell, Lowell, MA, United States; 2 Miner School of Computer and Information Sciences, University of Massachusetts Lowell, Lowell, MA, United States; 3 Department of Psychology, University of Massachusetts Lowell, Lowell, MA, United States

**Keywords:** causal inference, craniotomy, functional independence, rehabilitation timing, traumatic brain injury

## Abstract

Traumatic Brain Injury (TBI) affects approximately 69 million people globally each year and leaves over 5 million with lasting disability, making it a leading cause of death and long-term impairment across all ages. Yet, most TBI research still relies on correlation-based regressions and basic propensity score methods, which are insufficient for addressing treatment-selection bias. This limitation underscores the need for modern causal-effect models to produce actionable evidence. This work applies a unified causal inference framework to quantify the impact of craniotomy, rehabilitation timing, and rehabilitation intensity on cognitive, functional, and quality-of-life outcomes in moderate-to-severe TBI. Our approach integrates outcome-adaptive LASSO for confounder selection, causal graph neural networks for structure discovery, inverse-probability weighting for average treatment effects (ATEs), and a causal-effect variational autoencoder to account for latent confounding. We analyzed data from 79,604 patients in the U.S. Traumatic Brain Injury Model Systems (TBIMS) database. Key treatments included craniotomy, very-early versus delayed rehabilitation start, and short versus long rehabilitation stays. Outcomes included discharge Functional Independence Measure (FIM) cognitive and motor scores, as well as follow-up assessments of productivity, social participation, and life-satisfaction. Results showed that craniotomy was causally associated with modest but statistically significant reductions in all five discharge FIM domains (average ATE ≈ −0.10 to −0.17 on 1–7 scales). Very-early rehabilitation initiation was linked to improvements in follow-up productivity and life satisfaction (ATE≈ +0.03 to +0.09 on 0–1 scales). Longer rehabilitation stays yielded the largest positive effects, enhancing both follow-up productivity and global FIM scores (ATE ≈ +0.08 to +0.24). All models achieved ≥90% accuracy in treatment assignment prediction, supporting the strength of confounder control and the robustness of the causal inferences.

## 1 Introduction

Traumatic Brain Injury (TBI) remains a leading cause of death and long-term disability worldwide (1). Beyond its high incidence, TBI imposes a substantial clinical and societal burden, one that is further complicated by significant variability in patient outcomes and recovery trajectories (2). Management of moderate-to-severe TBI often includes both surgical and rehabilitation interventions (3). However, despite ongoing advances in these domains, patient outcomes remain variable and difficult to predict.

To address this uncertainty, researchers have increasingly turned to advanced statistical and machine learning methods. These tools aim to enhance our understanding of treatment efficacy, support individualized prognoses, and inform data-driven clinical decision-making in TBI care (4). Among the most consequential decisions in acute TBI management is whether to pursue surgical intervention, particularly craniotomy for hematoma evacuation or intracranial pressure control. Evidence from randomized trials and observational studies comparing craniotomy to conservative treatment remains mixed, with outcomes highly contingent upon patient selection, timing, and injury characteristics (5). Recent observational analyses using techniques such as propensity score matching and instrumental variable methods have suggested that, for carefully selected patients, craniotomy may yield cognitive benefits (6). However, further work is needed to elucidate how factors such as age and injury severity modulate these effects. The central challenge lies in identifying which patients are likely to benefit the most from surgical intervention, particularly in the context of complex confounding factors that influence both treatment decisions and outcomes (7–9).

Early initiation of rehabilitation has also emerged as a critical factor that influences long-term functional recovery. Agrawal and Joshi found that patients admitted to inpatient rehabilitation within days of injury achieved greater gains in Functional Independence Measure (FIM) scores both at discharge and one year post-injury (10). Specialized, multidisciplinary programs have similarly been shown to enhance recovery while reducing the length of hospital stay (11). Consequently, current clinical guidelines now recommend early rehabilitation for patients with disorders of consciousness (12). Moreover, family involvement has been associated with improved patient outcomes (13).

Rehabilitation intensity, often reflected by the overall length of stay, has emerged as a key healthcare delivery pattern linked to long-term recovery. Semlyen et al. demonstrated that intensive, multidisciplinary rehabilitation yields greater FIM improvements and real-world independence compared to single-discipline care (14). Spivack et al. reported a dose-response relationship, where longer stays and higher treatment intensity led tp better motor, cognitive, and social outcomes (15). This relationship has been further validated by randomized controlled trials (RCTs), which show that programs providing four hours per day of therapy accelerate functional gains compared to two-hour programs, without significantly altering final recovery endpoints (16).

At the system level, extended stays often reflect both patient complexity and the application of comprehensive protocols. Notably, longer rehabilitation durations have been associated with greater gains, even among more severely disabled patients (17). Supporting these clinical observations, neurobiological studies suggest that sustained and structured stimulation enhances neuroplasticity, enabling neural reorganization and offering a mechanistic rationale for the observed benefits of intensive rehabilitation (18).

In this study, we utilize the Traumatic Brain Injury Model Systems (TBIMS) National Database (*n*= 79,604) to estimate the causal effects of key surgical and rehabilitation decisions on patient recovery outcomes. Specifically, we investigate two direct clinical interventions, craniotomy decisions and the timing of rehabilitation initiation, and one healthcare delivery exposure, rehabilitation intensity, to provide a comprehensive understanding of both modifiable and systemic influences on recovery. To achieve this, we implement a unified causal inference framework that integrates several advanced methods: Outcome Adaptive Lasso (OAL) for confounder selection, Causal Graph Neural Network (CGNN) for causal structure discovery, Inverse Probability Weighting (IPW) for effect estimation, and Causal Effect Variational Autoencoder (CEVAE) for addressing latent confounding. This machine learning-enhanced approach is designed to overcome limitations of traditional causal inference techniques by capturing nonlinear treatment-outcome relationships and accounting for unmeasured confounding through rigorous bootstrap validation.

The key contributions of our work are:

1. We present a unified AI-enabled causal inference framework—integrating outcome-adaptive LASSO for confounder selection, causal graph neural networks (CGNN) for structure discovery, inverse probability weighting (IPW) for effect estimation, and a causal-effect variational autoencoder (CEVAE) to address latent confounding—to support robust treatment effect estimation from observational healthcare data.
2. We apply this framework to a large, real-world dataset of 79,604 patients from the U.S. Traumatic Brain Injury Model Systems (TBIMS), enabling scalable causal analysis across multiple domains of TBI care.
3. We generate high-fidelity actionable estimates of the causal effects of craniotomy, rehabilitation timing, and rehabilitation intensity on functional, cognitive, and quality of life outcomes, supporting future integration into AI-driven clinical decision support systems.

**Figure 1:**
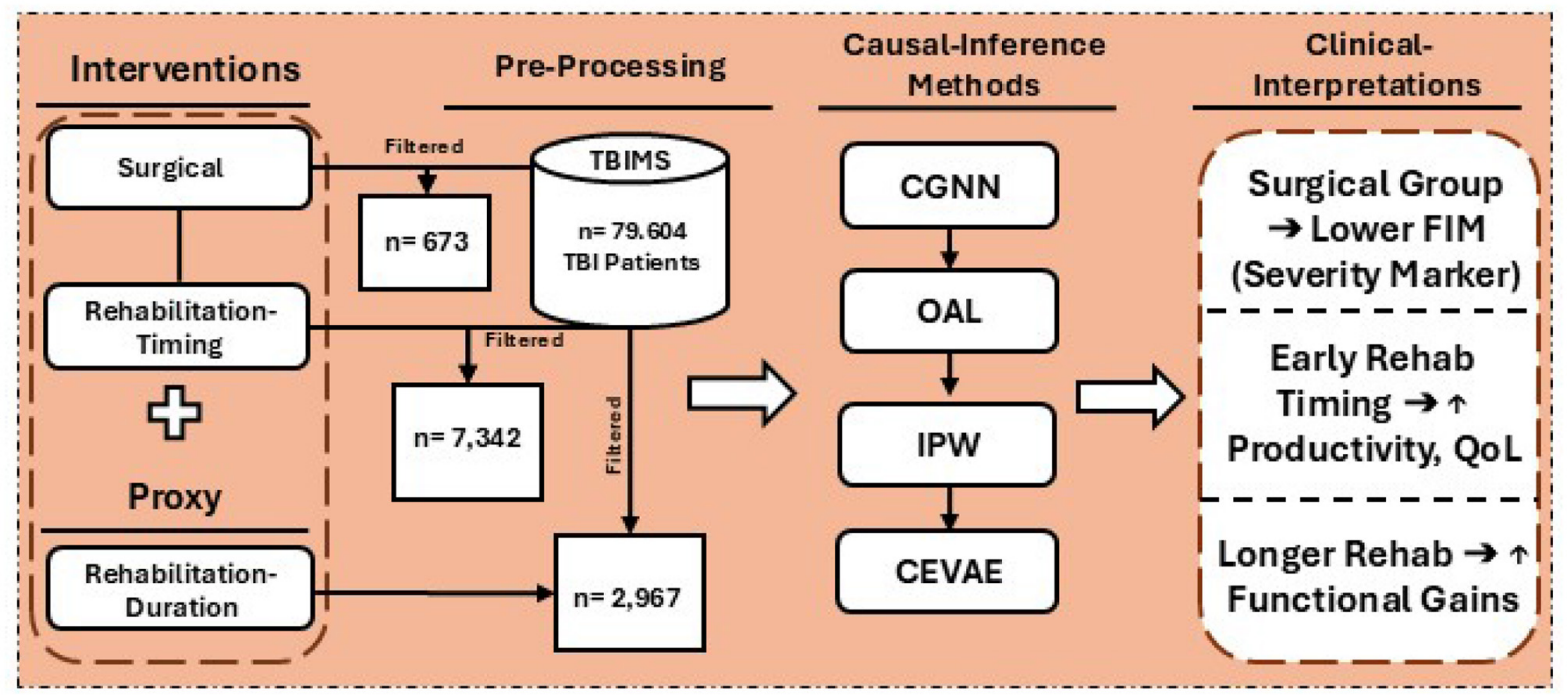
Overview of the study workflow, including data extraction from the TBIMS national database, categorization into surgical and rehabilitation cohorts, application of causal inference methods (CGNN, OAL, IPW, CEVAE), and clinical interpretation of effects on functional outcomes such as FIM scores, productivity, and quality of life.

This study builds on and advances prior work in TBI-related causal inference by incorporating cutting-edge machine learning methods designed to address key methodological limitations—particularly challenges in modeling nonlinear associations and accounting for latent confounders—that may not be fully addressed by conventional propensity score and instrumental variable approaches (19, 20). By systematically combining OAL, CGNN, IPW, and CEVAE, we demonstrate a scalable and robust analytical framework for large-scale healthcare data analysis. The study also provides a foundation for integration within AI-driven clinical decision support systems (AI-CDSS) to guide individualized treatment planning for TBI care. This integrated approach supports a more precise and individualized understanding of intervention effects, fills a critical gap in machine learning-enhanced causal inference within TBI research, and generates high-fidelity evidence to inform both personalized TBI care strategies, as well as system-level decision-making in biomedical informatics. The workflow of this study, including the data pipeline, intervention groups, and causal inference modeling strategies, is illustrated in Figure 1.

## 2 Materials and methods

### 2.1 Database description

The Traumatic Brain Injury Model Systems (TBIMS) National Database is a cornerstone resource for research on moderate-to-severe traumatic brain injury (TBI) in the United States. Established in 1987 and managed by the Traumatic Brain Injury National Data and Statistical Center (TBINDSC), the TBIMS collects comprehensive data through a network of federally funded rehabilitation centers. This database captures a wide array of clinical, demographic, and functional information, beginning at hospital admission and extending through inpatient rehabilitation and long-term community reintegration (21). Participants are enrolled based on standardized clinical criteria, including moderate-to-severe TBI confirmed by Glasgow Coma Scale scores, neuroimaging findings, or the duration of post-traumatic amnesia. The TBIMS cohort reflects a broad demographic and geographic distribution, representative of the diverse populations served by participating centers across the country. Data collection is longitudinal, with follow-up assessments occurring at one, two, and five years post-injury, and additional evaluations every five years thereafter. This structure enables robust analysis of recovery trajectories and long-term outcomes. The standardized variables include patient demographics, injury characteristics, acute care details, rehabilitation variables, and a range of functional and quality-of-life outcomes. All data are de-identified and collected in accordance with institutional and federal guidelines, ensuring participant privacy and compliance with ethical standards. The TBIMS National Database thus provides a unique and powerful platform for investigating the effects of clinical interventions and healthcare delivery patterns on recovery after moderate-to-severe TBI, supporting both observational studies and the development of advanced analytic approaches in rehabilitation research. This study used fully de-identified, publicly funded data from the TBIMS National Database, accessed with approval from TBINDSC, and was determined to be ‘Not Human Subjects Research' and exempt from IRB review under University of Massachusetts Lowell policy. The TBIMS dataset request was approved and fulfilled on January 25, 2025, when the de-identified data were received from the TBINDSC administrator.

### 2.2 Study population

Individuals were drawn from the Traumatic Brain Injury Model Systems (TBIMS) National Database and had sustained moderate-to-severe traumatic brain injury. Eligible participants were ≥16 years of age, admitted to a TBIMS-affiliated acute-care hospital within 72 hours of injury, and fulfilled established clinical criteria for moderate-to-severe TBI based on Glasgow Coma Scale score, duration of post-traumatic amnesia, or neuroimaging findings. After acute management, patients were transferred to a designated TBIMS inpatient rehabilitation center. The cohort spans diverse demographic and geographic backgrounds, reflecting the catchment areas of participating centers across the United States. Prospective data collection commenced during acute hospitalization and continued through inpatient rehabilitation and long-term follow-up. Written informed consent was obtained from all participants or their legally authorized representatives, and all records were fully de-identified in compliance with institutional and federal regulations.

### 2.3 Variables and measurements

**Figure 2:**
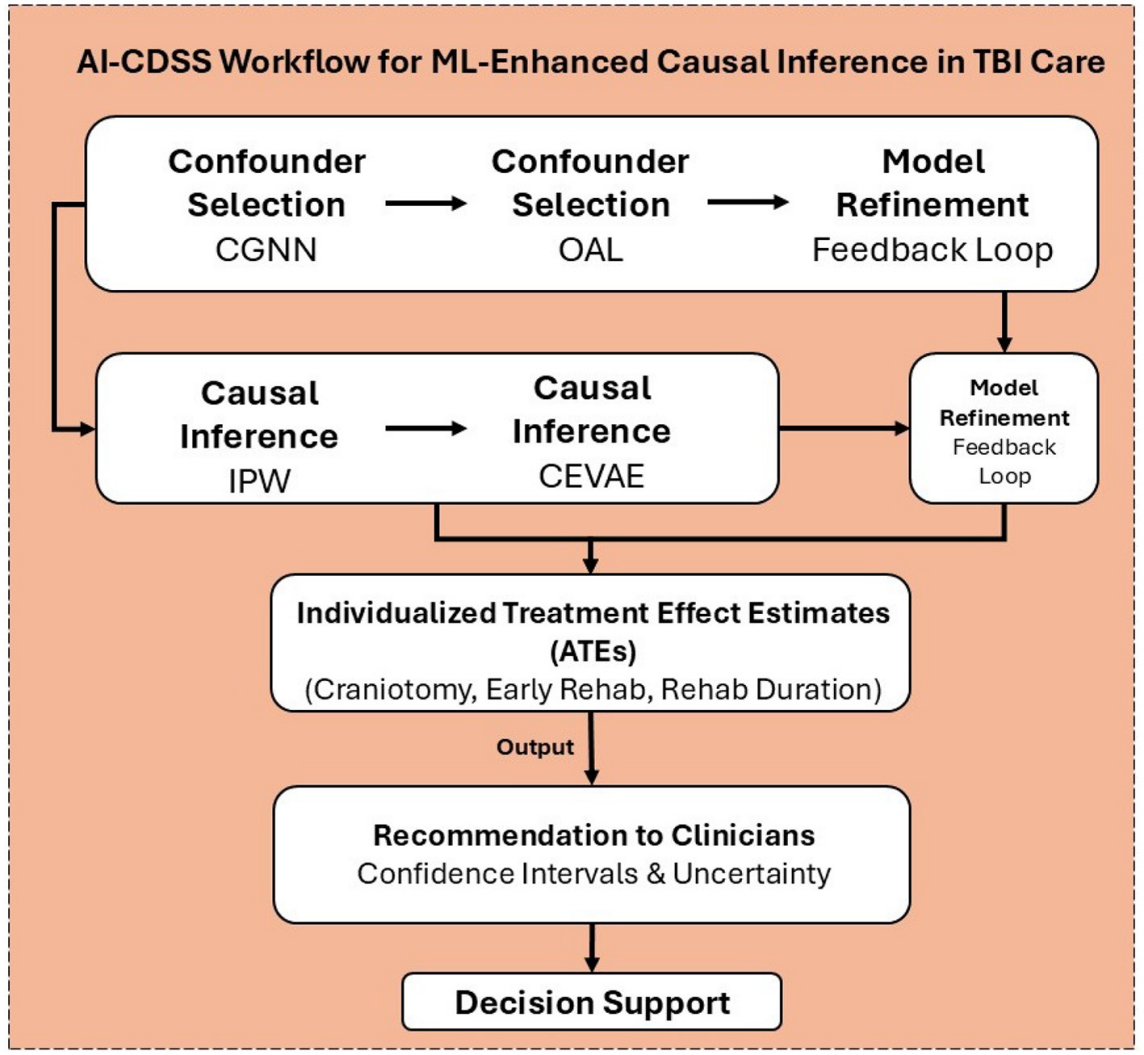
The pipeline integrates confounder selection (Outcome Adaptive Lasso, Causal Graph Neural Networks), causal inference (Inverse Probability Weighting, Causal Effect Variational Autoencoder), and model refinement within a feedback loop to generate individualized treatment effect estimates (ATEs) for interventions such as craniotomy, early rehabilitation, and rehabilitation duration. These estimates, along with confidence intervals and uncertainty measures, can guide data-driven recommendations within AI-driven clinical decision support systems for personalized TBI care.

The Traumatic Brain Injury Model Systems (TBIMS) National Database systematically captures an extensive array of standardized variables reflecting the clinical, demographic, and functional profiles of individuals with moderate to severe traumatic brain injury. Data acquisition commences at acute hospitalization and extends longitudinally through inpatient rehabilitation and long-term follow-up, enabling robust tracking of patient trajectories. Core variables encompass demographics (age, sex, race/ethnicity, education, employment), detailed injury characteristics (mechanism, Glasgow Coma Scale score, duration of loss of consciousness, post-traumatic amnesia), and acute care parameters (admission/discharge dates, length of stay, payor source). The database further includes granular rehabilitation data, such as admission and discharge scores on the Functional Independence Measure (FIM), rehabilitation length of stay, and discharge disposition. Participants undergo follow-up evaluations at one, two, five, ten, and fifteen years post-injury, with additional assessments every five years thereafter, capturing outcomes related to functional status, community participation, quality of life, re-hospitalization, and psychosocial adjustment. Standardized instrument, including the FIM, Satisfaction With Life Scale (SWLS), and the Ohio State University TBI Identification Method are employed to ensure consistency and validity across sites. Data collection utilizes medical record abstraction, structured interviews, and validated self-report questionnaires, all governed by rigorous operational definitions, coding protocols, and quality assurance procedures detailed in the TBIMS Data Dictionary, thereby ensuring high reliability and comparability across participating centers. The multi-step data processing and analysis pipeline built upon these variables is illustrated in Figure 2.

### 2.4 Cohort construction and pre-processing

A comprehensive data integration and pre-processing strategy was implemented to construct the analytic cohort for this study. The initial dataset encompassed multiple core data sources and accompanying code definition files containing variable metadata and coding schemes. A two-stage merging process was employed to unify clinical, demographic, and outcome variables across all records, resulting in a dataset comprising 687 variables and 79,604 observations, totaling approximately 28 million data points. To address the extensive use of special codes denoting various forms of missingness, all placeholder values indicating non-collected data, participant refusals, logical skips, or unknown entries were systematically recoded as missing (NaN). This harmonization step affected over 11 million data points across the integrated dataset. Subsequently, a structured variable retention protocol was applied to optimize data quality and analytic utility. Each variable was assigned a composite retention score, reflecting both completeness (the proportion of non-missing values) and data quality (the proportion of valid, non-placeholder entries). Variables were excluded if they exhibited more than 60% missingness, low data quality (greater than 90% invalid or placeholder values), redundancy, or lacked clinical relevance based on expert review. This rigorous filtering process reduced the number of variables from 687 to 352, retaining over half of the original features while ensuring robust clinical representation and minimizing potential sources of bias.

#### 2.4.1 Surgical intervention cohort

Following the initial data cleaning and variable filtering, we conducted intervention-specific pre-processing to create the surgical analysis dataset. From the cleaned cohort of 79,604 patients and 352 variables, we identified a subset of individuals who met the criteria for surgical decision-making analyses, specifically those with documented craniotomy decisions and complete outcome data at discharge. This filtering yielded a surgical intervention cohort comprising 673 patients, each with complete data on 5 primary cognitive and functional outcomes based on the Functional Independence Measure (FIM) scales at discharge.

#### 2.4.2 Rehabilitation-timing intervention cohort

For this phase, we applied scenario-specific filtering criteria to the preprocessed dataset of 79,604 patients. We implemented an “extreme strategy” to maximize clinical contrast and statistical power. We compared patients who received very early rehabilitation initiation ( ≤ 4.5 days post-injury) against those with delayed initiation (≥45 days post-injury). This approach excluded patients in the intermediate timing range (5–44 days) to create the clearest possible distinction between immediate versus delayed rehabilitation protocols. Binary contrast design was employed for compatibility maintenance with the causal inference framework where all models are defined on two levels of treatment. Methods such as Outcome Adaptive Lasso (OAL), Inverse Probability Weighting (IPW), and Causal Effect Variational Autoencoder (CEVAE) are defined for binary exposures and do not operate natively for multi-category or continuous treatments. This setting created a clear contrast between immediate and delayed rehabilitation and improved model stability. Excluding 72,262 patients in the intermediate category (5-44 days) sacrificed generalizability but provided uniform estimation across methods.

The rehabilitation-specific sample selection criteria included: (1) availability of rehabilitation admission timing data (DAYStoREHABadm), (2) complete outcome measurements for at least one primary endpoint (social participation, productivity, or quality of life domains), and (3) sufficient confounder data for causal adjustment. From the 79,604 patients, 7,342 met the extreme timing criteria. This group included 4,620 patients who received very early rehabilitation ( ≤ 4.5 days) and 2,722 patients who received delayed rehabilitation (≥45 days). This distribution reflects real-world clinical practice, where immediate rehabilitation is less common due to acute care requirements and medical stability considerations.

#### 2.4.3 Rehabilitation-intensity exposure cohort

For the rehabilitation intensity proxy analysis, we applied exposure-specific filtering to the pre-processed dataset of 79,604 patients to examine patterns of healthcare delivery, rather than direct clinical interventions. Unlike intervention analyses that focus on discrete decision points, this analysis targeted rehabilitation intensity as a proxy for patient complexity, recovery trajectory, and healthcare system characteristics. To operationalize rehabilitation intensity, we used an extreme contrast strategy that compared patients with short versus long rehabilitation stays. Individuals with moderate-length stays (15–30 days) were excluded to maximize the contrast between minimal and extended rehabilitation exposure. Following the extreme contrast strategy applied in the rehabilitation-timing cohort, this phase used a binary setup to distinguish patients with short and long rehabilitation stays. The causal inference framework relies on binary treatment variables, and models are not designed for multiple exposure levels. This approach focused on the most distinct patterns of care delivery and enhanced stability in estimating causal effects. A total of 76,637 patients with moderate-length stays (15–30 days) were excluded. The remaining groups represented the most distinct clinical and statistical contrasts for analysis.

The exposure-specific inclusion criteria for this analysis were:

- Availability of rehabilitation length of stay data,
- Completion of rehabilitation (excluding transfers and early discharges),
- Complete outcome measurements across five functional domains, and
- Sufficient healthcare system and patient-level data to allow for confounding control.

Applying these criteria yielded 2,967 cases with complete rehabilitation duration and covariate data, comprising 166 variables: 1 treatment, 5 outcomes, and 160 candidate confounders. The final sample included 1,718 patients with short stays (57.9%) and 1,249 with long stays (42.1%). These proportions reflect real-world delivery patterns; short stays may indicate rapid recovery or systemic constraints, while long stays often suggest greater clinical complexity or access to more comprehensive care protocols.

### 2.5 Multi-step average treatment effect estimation

To estimate average treatment effects (ATE) from high-dimensional, observational TBI data, we developed a three-step pipeline that integrates confounder selection, causal structure discovery, and effect estimation using both traditional and deep learning-based methods. This hybrid approach enhances robustness by addressing both measured and latent confounding through methodological triangulation.

**Step 1: confounder selection:** We employed a structured two-step approach that combined Outcome Adaptive LASSO (OAL) and Causal Graph Neural Networks (CGNN). OAL was first applied to generate outcome-specific confounder sets using regularized regression. These sets were then refined with CGNN to uncover the underlying causal structure and verify directional relationships among covariates, treatment, and outcome. Initially, OAL provided outcome-specific confounder sets, mathematically defined as:


$$\begin{array}{l} {\hat{\beta }}_{OAL}\left(\lambda ,\gamma \right)=\arg\underset{\beta }{\min}L\left(\beta \right) \end{array}$$ (1)

where β is the vector of model coefficients, λ is the regularization parameter that controls the penalty strength, and γ is the weighting factor that adjusts how strongly the adaptive weights affect each coefficient during estimation. To obtain the optimal coefficient estimates, the OAL method minimizes the loss function through regularized regression, as shown below:


$$\begin{array}{l} L\left(\beta \right)=\underset{i}{\sum }{\left(y_{i}-x_{i}\beta \right)}^{2}+\lambda \underset{j}{\sum }w_{j}|\beta_{j}| \end{array}$$ (2)

where *y*_*i*_ is the observed outcome for individual *i*, and *x*_*i*_ is the vector of predictors. The parameter λ controls the penalty applied to less important variables, and *w*_*j*_ represents the adaptive weight that scales the penalty for each coefficient β_*j*_.

**Step 2: structure discovery:** We optimized the gamma convergence factor (γ = 2) and lambda (λ) values (ranging from 10^−15^ to 10^0^) through cross-validation. This yielded 55-60 relevant confounders per outcome. We then implemented Causal Graph Neural Network (CGNN) with 1,000 bootstrap iterations to validate and refine the OAL selected variables. CGNN is an advanced deep-learning approach designed to uncover causal relationships from observational data. It utilizes neural networks structured around Directed Acyclic Graphs (DAGs) to estimate both the causal structure and the relationships simultaneously. Formally, CGNN represents variables *X*_*i*_ through neural network functions *f*_*i*_:


$$\begin{array}{l} X_{i}=f_{i}\left(\text{Pa}\left(X_{i}\right),\epsilon_{i}\right),\;i=1,\ldots ,n \end{array}$$ (3)

where Pa(*X*_*i*_) denotes the parent variables of *X*_*i*_, and ϵ_*i*_ is an independent noise component. CGNN effectively captures complex, nonlinear interactions between variables and achieved strong performance in our dataset (Training *R*^2^ = 97.04%, Test *R*^2^ = 92.84%).

**Figure 3:**
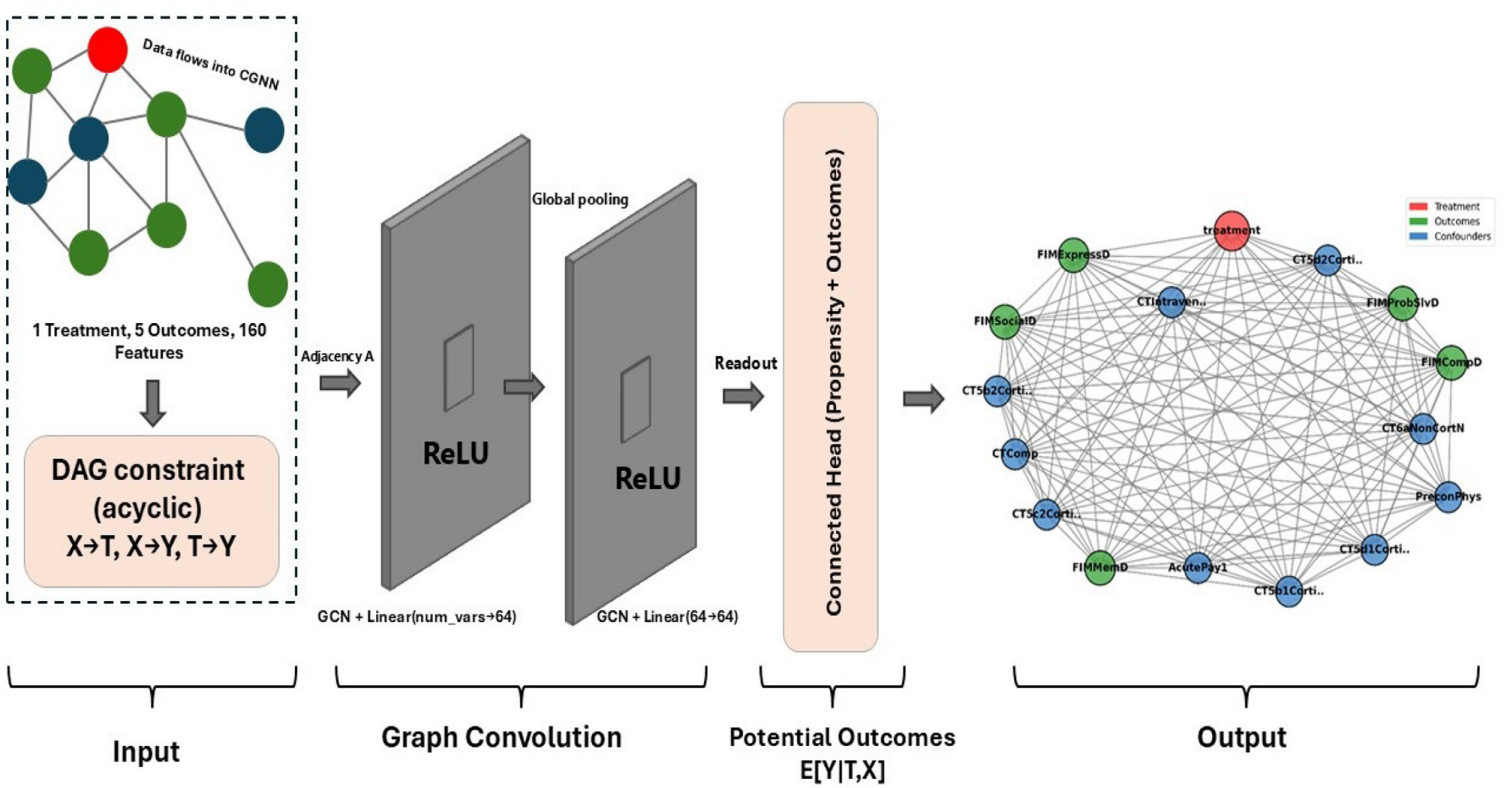
Graphical representation of the causal graph neural network (CGNN) framework, adjsuted for the surgical intervention scenario. The architecture consists of three main components: an input layer encoding one treatment variable (Required Craniotomy, red node), five cognitive and functional outcomes (green nodes), and 160 baseline covariates (blue nodes); a graph convolution module with directed acyclic graph (DAG) constraints enforcing acyclic information flow (*X*→*T*, *X*→*Y*, *T*→*Y*), two stacked graph convolution layers with ReLU activation, and a connected readout head estimating *E*[*Y*|*T, X*]; and an output layer representing learned causal dependencies among treatment, outcomes, and top ranked confounders. The top 10 confounders identified via Outcome Adaptive Lasso and CGNN include Pre-existing Physical Conditions (PreconPhys), Acute Payor Source (AcutePay1), CT compression (CTComp), Intraventricular Hemorrhage (CTIntraventricular), and cortical lesion locations detected on CT imaging: Right Parietal (CT5c2CorticalRPar), Left Occipital (CT5d1CorticalLOcc), Right Frontal (CT5b1CorticalRFront), Left Frontal (CT5a1CorticalLFront), Right Temporal (CT5b2CorticalRTemp), and Right Non-Cortical (CT6aNonCortN). Directed edges represent learned causal relations derived from adjacency matrix *A* and feature embeddings across graph convolution layers.

In simple terms, CGNN learns how variables are connected to each other in a network. Each variable, such as a patient's characteristic, treatment, or outcome, is represented as a node. The model passes information between these nodes through neural network layers that estimate how one variable may influence another. During training, CGNN builds a map of these connections, called an adjacency matrix, which shows the likely causal directions and strengths between variables. To keep the results interpretable, the network limits unnecessary connections and enforces a logical order where causes precede effects. The final output identifies the variables most strongly linked to treatment and outcomes. These variables represent the key confounders in the data. The structure of CGNN is illustrated in Figure 3.

While OAL identifies outcome-specific confounders through penalized regression, CGNN refines these selections by uncovering the underlying causal relationships between confounders and outcomes. By combining the strengths of OAL and CGNN, we reduced selection bias and retained only the most robust and clinically relevant confounders. To further improve model stability and avoid overfitting, we limited the final analysis to the top 25 confounders consistently identified across methods.

**Step 3: average effects estimation using latent confounding**. We applied two complementary causal inference methods: Inverse Probability Weighting (IPW) and Causal Effect Variational Autoencoder (CEVAE).

**Inverse probability weighting (IPW)**. uses propensity scores to assign weights to individuals, creating a pseudo-population where treatment is independent of confounders. For each individual *i*, the propensity score *e*_*i*_ is defined as:


$$\begin{array}{l} e_{i}=P\left(T_{i}=1|X_{i}\right) \end{array}$$ (4)

where *T*_*i*_ is the treatment indicator and *X*_*i*_ is the set of confounders. *P*(*T*_*i*_ = 1∣*X*_*i*_) represents the probability that individual *i* receives the treatment given their observed confounders. Accordingly, *e*_*i*_ denotes this estimated probability (the propensity score). *T*_*i*_ = 1 indicates treatment exposure, and *T*_*i*_ = 0 indicates no treatment. *X*_*i*_ includes all baseline variables that may influence both treatment assignment and outcomes. The weights are computed as:


$$\begin{array}{l} w_{i}=\{\begin{array}{ll} \frac{1}{e_{i}}, & \text{if }T_{i}=1\\ \frac{1}{1-e_{i}}, & \text{if }T_{i}=0 \end{array} \end{array}$$ (5)

The Average Treatment Effect (ATE) is then estimated by comparing the weighted outcomes between the treated and untreated groups. To ensure robustness, weights are trimmed at the 1st and 99th percentiles to handle extreme propensity scores. This method provides transparent and interpretable population-level estimates of ATE under the assumption of correctly measured confounders.

**Figure 4:**
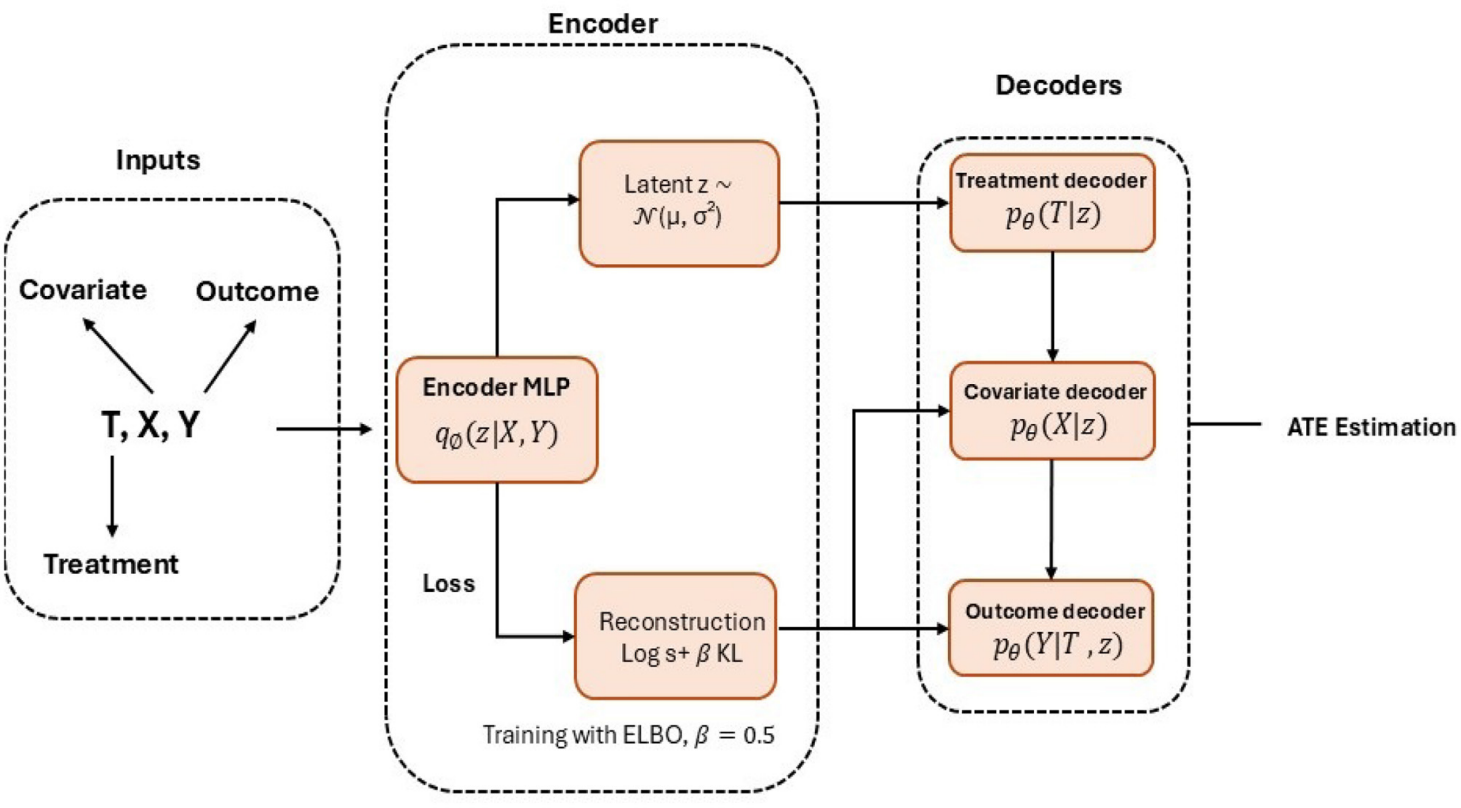
Graphical representation of the causal effect variational autoencoder (CEVAE) architecture. The framework consists of three main components: an input module that encodes observed covariates (*X*), treatment indicator (*T*), and outcomes (*Y*), representing the observed data generation process (*X*→*T*, *X*→*Y*, *T*→*Y*); an encoder network (*q*_ϕ_(*z*|*X, T, Y*)) that infers latent confounders $z\sim N\left(\mu ,\sigma^{2}\right)$ through a multilayer perceptron; and a decoder network (*p*_θ_) composed of three conditional branches–*p*_θ_(*T*|*z*), *p*_θ_(*X*|*z*), and *p*_θ_(*Y*|*T, z*) that reconstruct treatment assignment, covariates, and outcomes, respectively. Model training minimizes the evidence lower bound (ELBO) loss, defined as $L={𝔼}_{q_{\phi }\left(z|X,T,Y\right)}\left[\log p_{\theta }\left(X,T,Y|z\right)\right]-\beta \text{KL}\left(q_{\phi }\left(z|X,T,Y\right)||p\left(z\right)\right)$, with β = 0.5 controlling the regularization strength. The model estimates average treatment effects (ATE) as the difference between potential outcomes, *E*[*Y*|*T* = 1, *X*]−*E*[*Y*|*T* = 0, *X*].

**Causal Effect Variational Autoencoder (CEVAE)** is a deep generative model used to estimate causal effects in the presence of hidden confounders. It learns latent representations that reflect unobserved factors influencing both treatment and outcome. This helps improve the accuracy of estimated treatment effects. CEVAE models the data-generating process as:


$$\begin{array}{ll} z & \sim p\left(z\right), \end{array}$$ (6)

$$\begin{array}{ll} T & \sim p_{\theta }\left(T|z\right), \end{array}$$ (7)

$$\begin{array}{ll} X & \sim p_{\theta }\left(X|z\right), \end{array}$$ (8)

$$\begin{array}{ll} Y & \sim p_{\theta }\left(Y|T,z\right), \end{array}$$ (9)

where *z* represents latent confounders, and θ denotes model parameters. In this framework, *z* captures unobserved factors that influence both treatment assignment and outcomes, *T* is the treatment variable, *X* represents observed covariates, and *Y* denotes the outcome. The model assumes that each variable is generated conditionally based on its parents in the latent causal structure. This generative setup allows CEVAE to approximate hidden confounding effects by learning the joint distribution *p*_θ_(*z, T, X, Y*). The model is trained by maximizing the evidence lower bound (ELBO), with a modified loss function that balances reconstruction and regularization:


$$\begin{array}{l} L={𝔼}_{q_{\phi }\left(z|X,T,Y\right)}\left[\ell \left(z\right)\right] \end{array}$$ (10)

where L is the ELBO used as the training objective and *q*_ϕ_(*z*|*X, T, Y*) is the variational posterior distribution that approximates the true latent confounder distribution. ϕ represents the parameters of the encoder network and ℓ(*z*) is the inner loss term that measures how well the model reconstructs the observed data for a given latent variable *z*. Then we obtain the following equation for the inner loss function ℓ(*z*), which defines how the model learns from each latent representation.


$$\begin{array}{l} \ell \left(z\right)=\log p_{\theta }\left(X,T,Y|z\right)-\beta \text{ KL}\left(q_{\phi }\left(z|X,T,Y\right)||p\left(z\right)\right) \end{array}$$ (11)

where log*p*_θ_(*X, T, Y*|*z*) is the reconstruction term that measures how well the model reproduces the observed data given *z*. The term KL(*q*_ϕ_(*z*|*X, T, Y*)||*p*(*z*)) is the Kullback-Leibler divergence that regularizes the latent space by aligning the inferred distribution with the prior *p*(*z*). θ denotes the parameters of the decoder network, and β = 0.5 controls the trade-off between reconstruction accuracy and regularization. The architecture of the Causal Effect Variational Autoencoder (CEVAE) is shown in Figure 4.

Treatment effects are estimated using Monte Carlo dropout with 1000 samples to capture model uncertainty. This structure enables CEVAE to capture nonlinear dependencies and hidden confounding patterns through its deep learning architecture, improving causal effect estimation in complex clinical datasets. We selected IPW for its transparency and reliability in estimating ATE under measured confounding, with proper weight trimming to handle extreme propensity scores. CEVAE complements IPW by addressing potential unmeasured confounding and nonlinear relationships within the TBI dataset through its latent variable modeling. Together, these methods and our OAL-CGNN confounder selection pipeline improve the accuracy and reliability of treatment effect estimates by methodological triangulation.

To assess whether selecting 25 confounders introduced bias or instability, we performed sensitivity analyses across all three intervention domains (surgical, rehabilitation timing, and intensity) using 20, 25, and 30 confounders. Both inverse probability weighting (IPW) and augmented IPW (AIPW) were applied with 1,000 bootstrap iterations per analysis across 15 outcomes. Results demonstrated exceptional stability: IPW showed perfect consistency across all outcomes (15/15, 100%), and AIPW maintained high agreement (13/15, 86.7%). Combined, 93.3% of all estimates were concordant, with average treatment effects differing by less than ±0.001 and identical effect directions across all configurations. These findings confirm that the top 25 threshold provides an optimal balance between causal completeness and statistical efficiency, ensuring robust inference while mitigating risks of both under-adjustment and overfitting.

### 2.6 Analysis of interventions, exposures, and outcomes

We applied our multi-step causal inference framework (Section 2.5)—including confounder selection, causal structure discovery, and treatment effect estimation—to evaluate the effects of three clinically relevant intervention and exposure variables on recovery outcomes in individuals with moderate-to-severe TBI. These included (1) surgical intervention via craniotomy, (2) early vs. delayed rehabilitation timing, and (3) intensity of rehabilitation based on duration of the inpatient rehabilitation stay. Each analysis used an extreme binary contrast to maximize clinical interpretability and reduce misclassification. Outcome measures were selected based on clinical relevance, statistical robustness, and data availability.

#### 2.6.1 Surgical intervention analysis

We evaluated the causal impact of required craniotomy on cognitive and functional recovery at discharge among patients with moderate-to-severe traumatic brain injury. This investigation addressed the inherent complexities of observational surgical research, particularly the challenge of confounding by indication, through robust causal inference methods and validation strategies. By systematically accounting for the greater injury severity typically observed in patients selected for surgical intervention, the analysis provided nuanced insights into the short-term effects of craniotomy on recovery outcomes, thereby enhancing the clinical understanding of surgical decision-making in this high-risk population.

The primary treatment variable was Required_Craniotomy, defined as a binary indicator:


$$\begin{array}{l} \text{Required\_Craniotomy}=\{\begin{array}{ll} 1, & \;\text{if craniotomy was performed}\\ 0, & \;\text{otherwise} \end{array} \end{array}$$ (12)

This binary variable provides a clear distinction between surgical and non-surgical management. It simplifies the classification process and minimizes the risk of misclassification in acute TBI clinical decision-making.

We assessed five cognitive and functional outcomes at discharge using the Functional Independence Measure (FIM):

- **Comprehension (FIMCompD):** Verbal and written comprehension, essential for everyday functioning.
- **Memory (FIMMemD):** Short- and long-term memory, essential for cognitive recovery.
- **Social Interaction (FIMSocialD):** Social behavior and interaction quality, key to community reintegration.
- **Problem Solving (FIMProbSlvD):** Executive functioning and decision-making, vital for independent living.
- **Expression (FIMExpressD):** Verbal and non-verbal communication, critical for effective rehabilitation.

Each outcome was scored from 1 (complete dependence) to 7 (complete independence). Outcome data were complete for all 673 patients, ensuring robust and unbiased measurement.

#### 2.6.2 Early rehabilitation-timing analysis

We examined the causal effects of very early versus delayed rehabilitation initiation on functional, social, and quality of life outcomes in patients with TBI. The same causal inference methods were applied as the surgical intervention analysis, with multiple validation steps to address non-random treatment assignment and confounding by indication. We tested whether very early rehabilitation initiation ( ≤ 4.5 days post-injury, 5th percentile) compared to delayed initiation (≥45 days, 95th percentile) improves follow-up recovery in patients with moderate-to-severe TBI.

The primary treatment variable was rehabilitation timing, operationalized as a binary extreme-contrast indicator:


$$\begin{array}{l} \text{Rehabilitation\_Timing}=\{\begin{array}{ll} 1, & \text{Very Early,}\leq 4.5\text{ days}\\ 0, & \text{Late,}\geq 45\text{ days} \end{array} \end{array}$$ (13)

This strategy contrasting the 5th and 95th percentiles created a 10-fold difference in timing, minimizing ambiguity and confounding from moderate variations in initiation.

Outcome selection was determined through systematic data quality assessment and methodological requirements for robust causal inference. From the initial pool of 9 candidate outcomes (motor, cognitive, social, productivity, and quality of life domains at discharge and follow-up), we applied three technical selection criteria:

1. A minimum standard deviation of ≥0.15 on a normalized 0–1 scale to ensure meaningful effect estimation;
2. A missingness threshold of ≤ 30% to retain adequate statistical power without extensive imputation;
3. Successful convergence in preliminary OAL models, confirming model stability and interpretability.

These criteria ensured that selected outcomes were not only clinically meaningful but also supported robust causal modeling. Five follow-up outcomes met all criteria and were included in the final analyses, representing key dimensions of long-term recovery and community reintegration:

- **Productivity—participation domain (PART_Domain_ProdF):** Work and daily activities.
- **Life satisfaction (SWLSTOTF):** Subjective well-being.
- **Social functioning (PART_Domain_SocF):** Community and social relationships.
- **Social outcomes—Malec scale (Malec_SocialF):** Interpersonal functioning.
- **Productivity—Malec scale (Malec_ProdF):** Vocational and educational success.

All outcomes were normalized (0–1 scale) for comparability. This approach confirmed consistent interpretation across domains. Among 7,342 patients with extreme timing patterns (4,620 very early, 2,722 late), complete outcome data were available, supporting robust effect estimation. To address the Malec Scales, they are standardized assessment tools developed by James Malec to measure social integration and productive activity in individuals with brain injury (22, 23).

#### 2.6.3 Rehabilitation-intensity exposure analysis

We further examined the impact of rehabilitation intensity, operationalized as inpatient rehabilitation length of stay, to assess how healthcare delivery patterns influence TBI recovery. Unlike surgical or early rehabilitation decisions, duration of inpatient stay reflects system-level and policy factors beyond immediate clinical control.

This proxy-based analysis serves three critical methodological and clinical purposes: (1) it distinguishes modifiable clinical interventions from system-generated indicators, informing policy and protocol development; (2) it demonstrates the robustness and adaptability of our causal inference framework across different variable types from direct medical decisions to healthcare system patterns; and (3) it addresses a gap in TBI literature by evaluating how healthcare delivery characteristics independent of direct clinical decision-making influence functional and cognitive outcomes. By analyzing rehabilitation duration as a proxy exposure rather than a direct intervention, we extend our methodological scope and capture the influence of institutional and systemic variation on patient outcomes. This analysis completes the continuum from acute intervention through rehabilitation processes to long-term outcomes. We defined rehabilitation intensity using an extreme-contrast strategy:


$$\begin{array}{l} \text{Rehabilitation\_Intensity}=\{\begin{array}{cc} 1, & \text{Very Short,}\leq 14\text{ days}\\ 0, & \text{Very Long,}\geq 30\text{ days} \end{array} \end{array}$$ (14)

Five outcomes were selected through systematic screening based on two technical criteria: (1) high data completeness rates (>85%) in the follow-up cohort, and (2) comprehensive coverage of both objective functional domains (social participation, productivity) and subjective well-being measures. Outcome selection followed similar data quality standards as in the early rehabilitation analysis but was streamlined to two criteria due to reduced missingness and strong model convergence across all candidate outcomes. Two of these outcomes, Productivity—Participation Domain (PART_Domain_ProdF) and Productivity—Malec Scale (Malec_ProdF) were also analyzed in the previous intervention scenario to enable direct comparison across intervention types. Thus, the rest outcomes are as follows:

- **Motor function (FIMMOTF):** Physical mobility and daily activity performance.
- **Total function (FIMTOTF):** Combined motor and cognitive functional independence.
- **Cognitive function (FIMCOGF):** Communication, memory, and problem-solving abilities.

In summary, we applied a consistent multi-step causal inference framework to evaluate three distinct interventions and exposures across acute and post-acute care in individuals with moderate-to-severe TBI. Each analysis employed an extreme binary contrast, rigorous confounder selection, and robust effect estimation to isolate the impact of clinical and system-level factors on functional and quality-of-life outcomes. While the intervention types varied, all analyses leveraged the same methodological pipeline to ensure comparability and minimize bias. The results of these analyses are presented in the following section.

## 3 Results

### 3.1 Surgical intervention

We estimated the effects of craniotomy on discharge cognitive and functional outcomes using FIM domains: comprehension, memory, social interaction, problem solving, and expression. The analysis included 302 treated and 371 untreated patients. Across methods, craniotomy was associated with lower FIM scores in all domains. According to Table 1, OAL and IPW produced consistent, statistically significant negative estimates across all outcomes, with ATEs ranging from −0.10 to −0.17. CEVAE also showed negative effects, although with smaller absolute values, reinforcing the robustness of the findings. For comprehension and memory, the estimated effects ranged from −0.04 (CEVAE) to −0.15 (IPW), suggesting a decline in cognitive functioning post-craniotomy. For soical interaction and problem solving, the effects were similarly negative, with estimates between −0.07 (CEVAE) and −0.11 (IPW). The largest negative effects were observed for expression, where estimates ranged from −0.08 (CEVAE) to −0.17 (IPW). Craniotomy showed negative effects across all domains. OAL and IPW produced similar estimates. CEVAE showed smaller but still negative values. The adverse pattern persisted after adjusting for latent confounding. The agreement among methods suggests that lower outcomes in surgical patients likely reflect greater clinical complexity, not model bias.

**Table 1:** Treatment effects of craniotomy on discharge FIM outcomes.

**Outcome**	**Method**	**Treated**	**Untreated**	**ATE**	**95% CI lower**	**95% CI upper**	***P*-value**	**Bootstrap 95% CI**
FIMCompD	OAL	302	371	−0.1024^*^	−0.2004	−0.0044	0.0405	[−0.1620, −0.0494]
	IPW			−0.1496^*^	−0.1948	−0.0644	0.0010	[−0.1948, −0.0644]
	CEVAE			−0.0911^*^	−0.0927	−0.0894	< 0.001	[−0.0465, −0.0452]
FIMMemD	OAL	302	371	−0.1184^*^	−0.2164	−0.0204	0.0179	[−0.1495, −0.0451]
	IPW			−0.0921^*^	−0.1760	−0.0331	0.0080	[−0.1760, −0.0331]
	CEVAE			−0.0418^*^	−0.0430	−0.0405	< 0.001	[−0.0458, −0.0440]
FIMSocialD	OAL	302	371	−0.1140^*^	−0.2120	−0.0160	0.0226	[−0.1776, −0.0954]
	IPW			−0.1031^*^	−0.2076	−0.0530	0.0010	[−0.2076, −0.0530]
	CEVAE			−0.0701^*^	−0.0734	−0.0669	< 0.001	[−0.0865, −0.0847]
FIMProbSlvD	OAL	302	371	−0.1056^*^	−0.2036	−0.0077	0.0346	[−0.1651, −0.0799]
	IPW			−0.1093^*^	−0.1885	−0.0517	0.0020	[−0.1885, −0.0517]
	CEVAE			−0.0694^*^	−0.0714	−0.0669	< 0.001	[−0.0861, −0.0839]
FIMExpressD	OAL	302	371	−0.1300^*^	−0.2280	−0.0320	0.0093	[−0.2023, −0.1195]
	IPW			−0.1665^*^	−0.2291	−0.0846	0.0010	[−0.2291, −0.0846]
	CEVAE			−0.0796^*^	−0.0828	−0.0768	< 0.001	[−0.0609, −0.0579]

^*^Statistically significant at *p* < 0.05.

### 3.2 Rehabilitation-timing intervention

**Figure 5:**
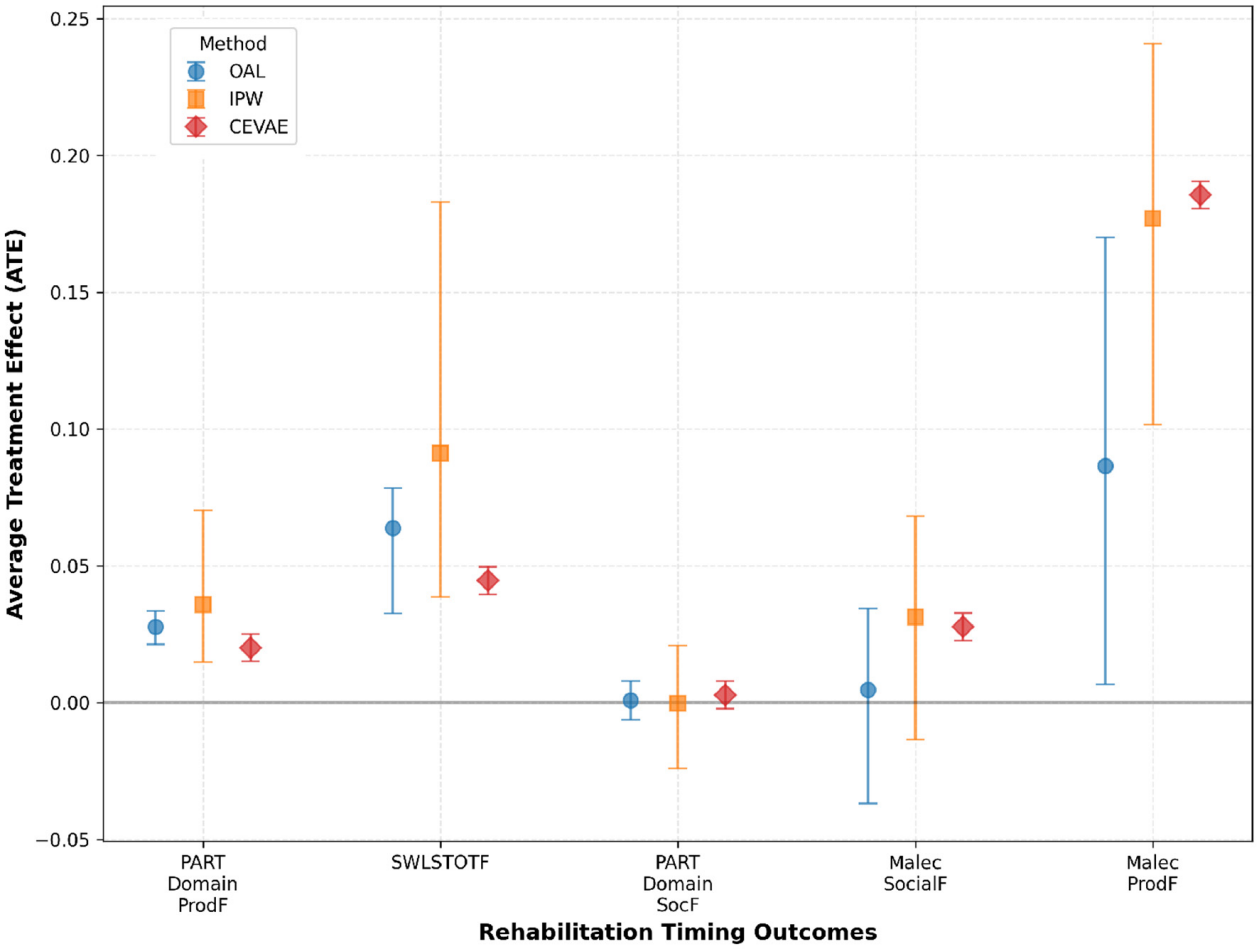
Estimated treatment effects and confidence intervals across multiple causal inference methods and outcome measures in the rehabilitation timing analysis. The results demonstrate consistent positive effects of very early rehabilitation initiation on productivity and life satisfaction, while findings for social outcomes are more variable across methods. These results align with the comparative analysis presented in Table 2.

Building on the methodological foundation, the OAL-CGNN pipeline identified 25 key confounders relevant to rehabilitation timing decisions. These included acute injury severity markers (Glasgow Coma Scale, CT findings), pre-injury functional status, acute care facility characteristics, and early medical complications. These confounders capture the clinical decision-making factors that determine when patients become medically stable for rehabilitation transfer. Across methods, very early rehabilitation timing showed positive effects on productivity and life satisfaction. As shown in Table 2, effects on social outcomes were mixed across methods, with some estimates indicating small or non-significant differences. For the Productivity - Participation Domain (PART Domain ProdF), OAL estimated an ATE of 0.03, IPW estimated 0.04, and CEVAE estimated 0.02, all statistically significant. For Life Satisfaction (SWLSTOTF), OAL estimated an effect of 0.06, IPW showed a higher effect at 0.09, and CEVAE estimated 0.04, consistently indicating improvements in perceived quality of life with earlier rehabilitation. Effects on social outcomes, including Productivity - Participation Domain (PART Domain ProdF) and Social Outcomes - Malec Scale (Malec SocialF), were small and varied. OAL and IPW estimates were near zero and non-significant, while CEVAE produced small but statistically significant positive estimates around 0.003 and 0.03, respectively. The results suggest potential benefits of early rehabilitation timing on functional recovery while indicating variability in social domain outcomes. These findings are visually summarized in Figure 5, which illustrates the estimated treatment effects and confidence intervals across causal inference methods and outcome domains.

**Table 2:** Treatment effects of very early vs. delayed rehabilitation timing on follow-up outcomes.

**Outcome**	**Method**	**Treated**	**Untreated**	**ATE**	**95% CI lower**	**95% CI upper**	***P*-value**	**Bootstrap 95% CI**
PART_Domain_ ProdF	OAL	4,620	2,722	0.0278^*^	0.0213	0.0336	< 0.001	[0.0213, 0.0336]
	IPW			0.0358^*^	0.0149	0.0703	0.0040	[0.0149, 0.0703]
	CEVAE			0.0200^*^	0.0199	0.0201	< 0.001	[0.0199, 0.0201]
SWLSTOTF	OAL	4,620	2,722	0.0638^*^	0.0325	0.0785	< 0.001	[0.0325, 0.0785]
	IPW			0.0912^*^	0.0387	0.1829	< 0.001	[0.0387, 0.1829]
	CEVAE			0.0447^*^	0.0445	0.0448	< 0.001	[0.0445, 0.0448]
PART_Domain_ SocF	OAL	4,620	2,722	0.0009	−0.0063	0.0080	0.8485	[−0.0063, 0.0080]
	IPW			−0.0002	−0.0239	0.0209	1.0000	[−0.0239, 0.0209]
	CEVAE			0.0028^*^	0.0027	0.0029	< 0.001	[0.0027, 0.0029]
Malec_SocialF	OAL	4,620	2,722	0.0047	−0.0368	0.0344	0.6800	[−0.0368, 0.0344]
	IPW			0.0313	−0.0135	0.0682	0.1560	[−0.0135, 0.0682]
	CEVAE			0.0277*	0.0275	0.0279	< 0.001	[0.0275, 0.0279]
Malec_ProdF	OAL	4,620	2,722	0.0866^*^	0.0067	0.1702	0.0200	[0.0067, 0.1702]
	IPW			0.1770^*^	0.1017	0.2408	< 0.001	[0.1017, 0.2408]
	CEVAE			0.1856^*^	0.1843	0.1869	< 0.001	[0.1843, 0.1869]

^*^Statistically significant at *p* < 0.05.

### 3.3 Rehabilitation-intensity exposure

We applied the established causal inference strategy to the rehabilitation-intensity analysis. This included adjustments specific to proxy variable estimation. Proxy exposures pose higher confounding risk than direct interventions. Using multiple methods allowed us to validate results under different assumptions. Applying a consistent framework across interventions and exposure enabled direct comparison of effect sizes and helped separate clinical decisions from systemic care patterns. The results in Table 3 represent consistent positive effects of longer rehabilitation stays across domains, with variations by method. OAL and IPW produced comparable positive estimates, while CEVAE provided additional insights under latent confounding assumptions. For the Productivity - Malec Scale (Malec ProdF), OAL estimated an ATE of 0.24, IPW estimated 0.22, and CEVAE estimated 0.20, all statistically significant. These values suggest that longer rehabilitation stays are associated with improved productivity outcomes at follow-up. For Motor Function (FIMMOTF) and Total Function (FIMTOTF), the effects were also positive, with OAL and IPW showing smaller estimates around 0.08–0.11, while CEVAE provided higher estimates, particularly for FIMMOTF at 0.17. This indicates that longer stays may have a beneficial impact on functional mobility and total FIM scores, though the size of these effects varies depending on the method used. For Cognitive Function (FIMCOGF), the estimated effects ranged from 0.06 (IPW) to 0.09 (CEVAE), suggesting potential cognitive benefits linked to longer rehabilitation durations. All estimators showed positive links between longer stays and better outcomes. Differences among methods were small. IPW and OAL produced similar results. CEVAE estimated slightly higher effects, possibly due to nonlinear adjustment of residual confounding. These findings support the same direction of effect but require caution in interpretation because of high treatment predictability and selection bias.

**Table 3:** Treatment effects of long vs short rehabilitation intensity on follow-up outcomes.

**Outcome**	**Method**	**Treated**	**Untreated**	**ATE**	**95% CI lower**	**95% CI upper**	***P*-value**	**Bootstrap 95% CI**
Malec_ProdF	OAL	1,718	1,249	0.2385^*^	0.2191	0.2607	0.010	[0.2191, 0.2607]
	IPW			0.2152^*^	0.1180	0.2686	0.010	[0.1083, 0.2730]
	CEVAE			0.2003^*^	0.1108	0.1634	< 0.001	[0.1108, 0.1634]
FIMMOTF	OAL	1,718	1,249	0.1136^*^	0.1011	0.1276	0.010	[0.1011, 0.1276]
	IPW			0.0829^*^	0.0594	0.1248	0.005	[0.0474, 0.1300]
	CEVAE			0.1691^*^	0.1249	0.1795	0.0107	[0.1249, 0.1795]
FIMTOTF	OAL	1,718	1,249	0.1068^*^	0.0975	0.1168	0.010	[0.0975, 0.1168]
	IPW			0.0829^*^	0.0677	0.1146	0.005	[0.0616, 0.1189]
	CEVAE			0.1503^*^	0.0962	0.1602	0.0118	[0.0962, 0.1602]
FIMCOGF	OAL	1,718	1,249	0.0753^*^	0.0666	0.0855	0.010	[0.0666, 0.0855]
	IPW			0.0631^*^	0.0409	0.0865	0.005	[0.0367, 0.0875]
	CEVAE			0.0934	0.0942	0.1431	0.2482	[0.0942, 0.1431]
PART_Domain_ProdF	OAL	1,718	1,249	0.0733^*^	0.0637	0.0843	0.010	[0.0637, 0.0843]
	IPW			0.0687^*^	0.0366	0.0895	0.005	[0.0333, 0.0926]
	CEVAE			0.0808	0.0537	0.0815	0.1789	[0.0537, 0.0815]

^*^Statistically significant at *p* < 0.05.

### 3.4 Treatment assignment modeling performance

We evaluated treatment assignment models across the three intervention/exposure analyses; craniotomy, rehabilitation timing, and rehabilitation intensity using Logistic Regression (LR), Random Forest (RF), Support Vector Machine (SVM), XGBoost, and Naive Bayes. These models were selected to represent a comprehensive range of algorithmic approaches: LR for its simplicity and statistical grounding, RF for handling complex non-linear relationships, SVM for its performance in high-dimensional settings, XGBoost for gradient boosting optimization, and Naive Bayes for probabilistic classification with independence assumptions.

**Model training process:** for each algorithm, we implemented a standardized training pipeline. Explanatory variables consisted of the same confounder sets used in causal inference analyses (25 common confounders identified by OAL-CGNN consensus). Response variables were binary treatment indicators: craniotomy (1 = yes, 0 = no), rehabilitation timing (1 = very early ≤ 4.5 days, 0 = late ≥45 days), and rehabilitation intensity (1 = short ≤ 14 days, 0 = long ≥30 days). Hyperparameter optimization was performed using 5-fold cross-validation with grid search for optimal parameters: LR (*C* = 1.0, L2 penalty), RF (*n*_*estimators* = 200, *max*_*depth* = 8), SVM (*C* = 1.0, RBF kernel), XGBoost (*learning*_*rate* = 0.1, *max*_*depth* = 6), and Naive Bayes (Gaussian priors). Performance evaluation used train-test split (70/30) with stratified sampling to maintain class balance.

- **Craniotomy:** XGBoost achieved the highest accuracy (0.970) and precision (0.964), while RF maintained strong performance across all metrics. Naive Bayes showed lower performance but provided probabilistic clarity (Table 4).
- **Rehabilitation timing:** XGBoost demonstrated superior performance with 0.987 accuracy and 0.990 F1-score, significantly outperforming other algorithms. RF remained competitive as the second best performer (Table 5).
- **Rehabilitation intensity:** XGBoost again achieved the highest accuracy (0.971) and F1-score (0.975), while RF and LR maintained strong performance. Naive Bayes showed reduced effectiveness in this domain (Table 6).

**Table 4:** Average treatment assignment prediction performance across models (surgical).

**Algorithm**	**Accuracy**	**Balanced accuracy**	**F1-score**	**Precision (treated)**	**Recall (treated)**
Logistic (L2)	0.912	0.915	0.907	0.868	0.949
Random forest	0.953	0.955	0.950	0.928	0.974
SVM	0.898	0.904	0.896	0.837	0.965
XGBoost	0.970	0.970	0.967	0.964	0.971
Naive Bayes	0.885	0.886	0.876	0.855	0.899
Average	0.924	0.926	0.919	0.894	0.952

**Table 5:** Average treatment assignment prediction performance across models (rehabilitation-timing).

**Algorithm**	**Accuracy**	**Balanced accuracy**	**F1-score**	**Precision (treated)**	**Recall (treated)**
Logistic (L2)	0.900	0.905	0.917	0.952	0.885
Random forest	0.964	0.962	0.971	0.973	0.969
SVM	0.928	0.929	0.942	0.959	0.925
XGBoost	0.987	0.985	0.990	0.988	0.991
Naive Bayes	0.815	0.764	0.867	0.791	0.959
Average	0.919	0.909	0.937	0.933	0.946

**Table 6:** Average treatment assignment prediction performance across models (rehabilitation intensity).

**Algorithm**	**Accuracy**	**Balanced accuracy**	**F1-score**	**Precision (treated)**	**Recall (treated)**
Logistic (L2)	0.901	0.904	0.925	0.954	0.897
Random forest	0.957	0.959	0.968	0.984	0.953
SVM	0.886	0.890	0.912	0.949	0.878
XGBoost	0.971	0.971	0.975	0.970	0.980
Naive Bayes	0.756	0.756	0.751	0.740	0.763
Average	0.894	0.896	0.906	0.919	0.894

The models consistently performed well across interventions, with XGBoost yielding the most reliable results across all three cohorts, followed by Random Forest. The high prediction accuracy (>90% average) across all algorithms confirms the strong confounding by indication present in treatment assignment decisions.

To more specifically quantify which of the baseline factors assigned treatment and to determine the presence of confounding by indication, we also conducted an extended feature importance analysis on six other complementary techniques. These were (1) F-Test (ANOVA) that quantifies statistical significance of treatment group mean differences. (2) Mutual Information: Detects non-linear associations between features and treatment allocation. (3) Random Forest importance: Quantifies feature importance through tree-based splits. (4) Logistic Regression coefficients: Quantifies linear contribution of each predictor to treatment prediction. (5) Recursive Feature Elimination (RFE): Successively removes less informative features. (6) Correlation Analysis: Quantifies strength of linear associations. As seen from Table 7, composite scores are the normalized and averaged importance of all six feature selection methods. From analysis, assignment of the comatose patients to treatment was mainly based on baseline severity variables, which included the Glasgow Coma Scale (GCS) components, Functional Independence Measure (FIM) admission scores, and Post-Traumatic Amnesia (PTA) duration.

**Table 7:** Three top predictors by intervention domain (averaged across six feature-selection methods).

**Domain**	**Variable (meaning)**	**Average (all methods)**
Surgical (craniotomy)	GCSMot (functional motor response)	94.7
	GCSVer (verbal response)	91.7
	DAYStoREHABadm (days to rehab admission)	82.6
Rehabilitation timing	PTADays (post-traumatic amnesia duration)	95.3
	AGENoPHI (age at injury)	84.0
	GCSMot (functional motor response)	83.3
Rehabilitation intensity	FIMExpressA (FIM Expression at admission)	96.0
	FIMCompA (FIM Comprehension at admission)	93.3
	PTADays (Post-traumatic amnesia duration)	92.7

## 4 Discussion

### 4.1 Clinical interpretations

#### 4.1.1 Surgical intervention

The analysis shows consistent, statistically significant negative effects of craniotomy across cognitive and functional domains. Results indicate that patients undergoing craniotomy experience moderately worse cognitive and functional outcomes at discharge compared to non-surgical patients, after controlling for confounders. Our comprehensive causal analysis demonstrates that, within a large, well-characterized cohort of moderate-to-severe TBI patients, required craniotomy is associated with worse cognitive and functional outcomes at discharge across all measured FIM domains. These associations are robust across multiple advanced causal inference frameworks (OAL, IPW, CEVAE) and remain consistent after rigorous confounder selection and validation.

However, these findings likely reflect confounding by indication where patients requiring surgical intervention present with more severe injuries that independently predict worse functional outcomes, despite comprehensive confounder adjustment. Our results align with established clinical literature demonstrating similar patterns. Multiple propensity-matched and meta-analytic studies have reported that patients undergoing craniotomy or Decompressive Craniectomy (DC) for TBI experience lower functional independence compared to those managed non-surgically. For example, Kelly et al. (24), focus specifically on adult patients with severe TBI, found that individuals who underwent Craniectomy had significantly greater impairment on the Glasgow Outcome Scale-Extended and were less likely to be employed at one-two years post-injury, while acknowledging that these patients had more severe initial presentations. This adult-focused finding aligns with our mixed adult-pediatric cohort results, though age-specific effects within our population warrant further investigation. Similarly, Guo et al. (6) in their propensity-matched study of 120 adult TBI patients (mean age 46-52 years), demonstrated that patients requiring more invasive procedures (Decompressive Craniectomy) had worse long-term outcomes compared to those needing craniotomy only, consistent with a severity gradient where more invasive interventions are reserved for more critically ill patients.

These adult-specific findings support our observations across a broader age range that includes both adult and pediatric patients. Recent systematic reviews and meta-analyses confirm that surgical interventions can reduce mortality and intracranial pressure in severe TBI, though functional outcomes remain challenging to improve due to underlying injury severity. A 2025 meta-analysis (25), analyzing five randomized controlled trials predominantly involving adult TBI populations, found robust mortality reduction with surgical intervention but no significant improvement in functional outcomes and this highlighted the complex relationship between life-saving procedures and long-term recovery (26, 27). While these adult-focused trial results inform our findings, the inclusion of pediatric patients (ages 16+) in our TBIMS cohort provides broader generalizability across age groups. These findings confirm that while craniotomy and DC remain essential for managing life-threatening complications, the observed functional outcomes primarily reflect the severity of injury necessitating surgical intervention rather than adverse effects of surgery.

Clinically, our findings support the established understanding that patients requiring craniotomy represent a more severely injured population with inherently worse prognosis. The consistency of these associations across multiple causal inference methods suggests that even advanced statistical techniques cannot fully eliminate confounding by indication in observational surgical research. This reinforces the importance of multidisciplinary care models that recognize both the life-saving necessity of surgical intervention and the need for intensive, long-term rehabilitation support for these high-severity patients. Our study adds to the existing evidence on the difficulties of achieving functional recovery after surgical management of TBI and highlights the importance of comprehensive post-acute care plans that address the needs of this complex patient group.

#### 4.1.2 Rehabilitation-timing intervention

The analysis reveals statistically significant but clinically marginal associations between very early rehabilitation timing and some follow-up outcomes. While productivity measures showed small positive associations across methods (0.02–0.19 points), the clinical significance of these differences remains unclear given the absence of established minimal clinically important differences for these outcome scales. The largest effects were observed for 'Productivity—Malec Scale (Malec ProdF)' (0.09–0.19 points), which may represent the only potentially meaningful clinical difference, though this requires validation against functional benchmarks. These modest effect sizes are consistent with recent systematic reviews that have noted the heterogeneity in rehabilitation timing benefits and the need for more nuanced interpretation of statistical versus clinical significance in adults of working age, a population broadly similar to our primarily adult TBIMS cohort (28, 29).

Social, participation, and productivity outcomes provided contradictory results by approaches. Conventional causal approaches (OAL, IPW) estimated small or no effects for social participation domains. CEVAE found moderate positive correlations. These differences arise from how each model is built. IPW conditions on ignorability and uses measured covariates only. It is sensitive to missing or crude covariates. OAL discourages weak predictors and shrinks small effects to zero. CEVAE incorporates latent layers that could capture hidden or nonlinear patterns. CEVAE could overfit or be ill conditioned. When CEVAE showed effects not seen in IPW or OAL, they likely reflected unobserved or nonlinear confounding. CEVAE results were taken as exploratory. More reliance was placed on effects seen in two or more estimators. This pattern concurs with multicenter studies which show that social reintegration outcomes are less frequently affected by intervention timing compared to functional domains. Such heterogeneity likely reflects psychosocial impacts that transcend the timing of rehabilitation in adults with TBI, a group similar to our dataset (30, 31). The strong prediction success of treatment (93% average) suggests prevailing selection effects. Rehabilitation timing decisions appear extensively organized and guided by patient factors.

Such systematic assignment patterns suggest substantial confounding by indication that may not be fully addressed by current adjustment methods, potentially limiting the causal interpretation of observed associations. This finding is consistent with observational studies demonstrating that rehabilitation timing is heavily influenced by injury severity, medical complications, and institutional factors of adults with TBI (32, 33). The findings suggest that timing effects, if present, are considerably smaller than selection effects in determining rehabilitation outcomes. The results emphasize the importance of individualized clinical decision-making that prioritizes medical readiness and appropriate care transitions.

#### 4.1.3 Rehabilitation-intensity proxy

The analysis reveals positive associations between longer rehabilitation duration and functional outcomes across multiple domains, with effect sizes ranging from 0.06 to 0.24 points. However, the exceptionally high treatment prediction accuracy (91.5%) suggests that rehabilitation duration is almost entirely determined by patient characteristics, which limits the causal interpretation of these associations. Patients requiring longer rehabilitation stays likely present with greater injury severity, medical complications, and baseline functional impairments, factors that independently influence both length of stay and recovery trajectories.

While these positive associations are consistent with dose response hypotheses observed in controlled rehabilitation studies, such as those by Spivack et al. (15) and Zhu et al. (16), it is important to contextualize our findings. Prior work has typically focused on adult TBI populations aged 18-65 years under more controlled settings, whereas our broader cohort includes patients with a wider range of clinical complexity and system exposure. As such, the observed associations more likely reflect confounding by indication, where rehabilitation duration acts as a proxy for underlying patient needs and complexity, rather than a modifiable intervention. Previous research has shown that discharge timing from acute care hospitals is only partially explained by clinical factors. Sorensen et al. (34) found that for adults aged ≥18 years treated at Level I trauma centers, only about 20% of discharge timing was attributable to clinical variables; the remainder was driven by administrative, insurance, and system-level determinants that reflect patient acuity and institutional practices.

Additionally, the clinical significance of the observed effect sizes remains uncertain in the absence of established minimal important differences for the functional outcome measures used. Thus, while extended rehabilitation may benefit certain patients, it cannot be assumed that artificially extending stays would improve outcomes. Healthcare systems should instead focus on ensuring that rehabilitation duration is tailored to individual patient needs. Evans et al. (35) studied older adults (≥65 years) discharged to skilled nursing facilities, a population distinct from the broader adult cohort analyzed in our study. Similarly, Avesani et al. (36) examined a younger adult TBI cohort (mean age 43.6 years) and found that acute-care length of stay and admission FIM scores, rather than demographic factors, independently predicted rehabilitation duration. These findings highlight the strong influence of patient complexity on stay length. Future research should aim to establish clinically meaningful thresholds for rehabilitation duration and determine whether observed outcome differences persist after rigorous adjustment for injury severity, recovery potential, and healthcare system variables.

## 5 Conclusion

We systematically evaluated associations between surgical intervention, rehabilitation timing, and rehabilitation duration with cognitive, functional, and quality-of-life outcomes across four distinct intervention domains. Across all analyses, we observed consistently high treatment prediction accuracy: 93.1% for rehabilitation timing, 91.5% for rehabilitation duration, and 92.1% for surgical intervention. These high accuracies suggest that observed associations likely reflect confounding by indication rather than true causal treatment effects.

Surgical interventions were negatively associated with functional outcomes, a finding that likely reflects underlying injury severity among patients requiring surgery, rather than the impact of surgery itself. Similarly, while rehabilitation timing may represent a modifiable intervention and rehabilitation duration a proxy for care intensity, the high treatment prediction accuracy of these variables from patient covariates suggests that both are more likely influenced by baseline patient complexity and care needs.

Past research shows that minimal clinically important differences (MCIDs) for the Functional Independence Measure (FIM) are typically 17-22 points for total scores and 4-5 points for domain-level changes, depending on population and injury severity (37). In contrast, the ATEs estimated in this study (0.06-0.24 points) are statistically significant but unlikely to be clinically meaningful. The TRACK-TBI cohort found that about half of patients with severe TBI regained functional independence within one year (38). This finding opens up wide variation in recovery outcomes. Systematic reviews also present that motor and cognitive rehabilitation often produce measurable but modest improvements (39, 40). An old work by Whitlock and Hamilton (41) found that few patients achieved full independence at discharge. Those who improved showed only small gains in FIM scores.

A practical pathway toward clinical deployment can be addressed in three stages. First, individualized treatment effect models should be integrated into an interactive dashboard that displays effect estimates with confidence intervals and represents uncertainty through probabilistic ranges rather than fixed point values. Second, each recommendation should include the most influential patient or clinical variables and clear indicators when the model depends on extrapolated or weakly supported data regions. Third, prospective validation across multiple rehabilitation centers is required to assess usability, clinician confidence, and the influence of model based guidance on treatment planning and patient outcomes. These steps provide a structured path for advancing the current causal framework from an analytic research tool to a system that supports evidence based clinical decision making.

Based on this translational framework, broader implications follow for their integration into AI-facilitated clinical decision support. The proposed methodological framework has ready implications for implementation in AI-based clinical decision support systems (AI-CDSS) for the management of TBI. By applying patient specific covariate profiles, models herein can calculate individualized treatment effect estimations, particularly for surgery planning and rehabilitation timing. This offers the possibility of enabling more informed, data driven advice and exchange among patients, families, and clinicians. Operationalization is guarded, however, the very high treatment predictability observed in our study underscores the contribution of patient complexity to treatment allocation. Prospective real-world clinical workflow validation will be important. Moreover, the ensuing AI-CDSS tools developed on this approach must place utmost emphasis on transparency, control by clinicians, and ethical protections to facilitate that decision support acts as an additive and not a substitute for expert clinical judgment in TBI treatment. Key ethical principles are algorithmic transparency, risk of bias in training data, equitable access to AI-CDSS tools, and arrangements for monitoring responsible use in practice.

## 6 Limitations and future work

Limitations of this study include the inability to fully address unmeasured confounding, despite the use of advanced methodological approaches, as well as the observational nature of the data, which inherently limits causal inference due to selection effects. Additionally, outcome measurement was limited to discharge and early follow-up assessments, without extended longitudinal tracking or comprehensive patient-reported outcomes.

Although several findings reached statistical significance, many of the observed average treatment effects (ATEs) were modest. These effect sizes may not translate into meaningful changes at the individual patient level and may fall below thresholds considered clinically impactful. As a result, while our findings provide valuable insight into potential treatment-outcome associations, their standalone clinical utility remains limited without further validation.

Another limitation is that the existing causal framework only simulates treatment assignment and outcomes according to baseline covariates. Realistically, recovery following traumatic brain injury is a process that changes over time while patients' neurological, metabolic, and systemic states develop between acute admission and rehabilitation initiation. Intermediate events such as infections, secondary neurological insults, or instability shifts may be time varying confounders impacting on the intensity or the timing of the rehabilitation and subsequent functional outcomes simultaneously. Because these dynamic factors were not modeled explicitly, residual bias on the basis of time dependent confounding cannot be ruled out with certainty. Future research should employ longitudinal covariate designs and advanced causal models such as marginal structural models or dynamic treatment regime modeling to better identify these changing patient pathways and increase causal validity in real-world rehabilitation settings.

Looking forward, future work will focus on testing and refining the proposed models using independent TBI datasets across diverse healthcare settings to enhance generalizability and reliability. Broader deployment within AI-driven Clinical Decision Support Systems (AI-CDSS) will also require ongoing monitoring for bias, fairness across demographic and clinical subgroups, interpretable outputs, and clinician-centered design to build trust, promote adoption, and mitigate disparities in treatment recommendations.

## Data Availability

The data analyzed in this study is subject to the following licenses/restrictions: The datasets analyzed in this study are available from the Traumatic Brain Injury Model Systems (TBIMS) National Database, maintained by the TBIMS National Data and Statistical Center. Access to the Public Use Dataset requires completion of a request form and a Data Use Agreement. More recent or customized data require formal approval by the TBIMS NDSC and the TBIMS Research Committee. Requests to access these datasets should be directed to: https://www.tbindsc.org/.
